# Associations between condylar height relative to occlusal plane and condylar osseous condition and TMJ loading based on 3D measurements and finite element analysis

**DOI:** 10.1038/s41598-024-80442-x

**Published:** 2024-11-22

**Authors:** Yanji Gong, Jinyi Zhu, Fangjie Zheng, Yunfan Zhu, Shangyan Sui, Yang Liu, Deqiang Yin

**Affiliations:** 1 National Clinical Research Center for Oral Disease, Department of Jinjiang Outpatient, West China Hospital of Stomatology, Sichuan University, Chengdu, 610041 China; 2 National Clinical Research Center for Oral Disease, Department of Temporomandibular Joint, West China Hospital of Stomatology, Sichuan University, Chengdu, 610041 China; 3 College of Aerospace Engineering, Chongqing University, Chongqing, 400044 China

**Keywords:** Condylar height, Temporomandibular point, Three-dimensional measurement, Three-dimensional finite element, Mandibular muscles, Cone-beam computed tomography

## Abstract

To investigate the relationship between condylar height relative to occlusal plane (CHO) and condylar osseous condition and the changes of condylar stress loading before and after CHO modifications. The condylar osseous conditions of 434 temporomandibular joints (TMJ) were assessed and grouped. Measurements of anatomical parameters were performed on CT-based reconstructed 3D stomatognathic models. Differences in anatomical parameters of the jaws in the different groups were compared, and the correlation between the Angle α (representing the CHO ratio) and related parameters was investigated. A finite element model (FEM) was constructed using 3D finite element analysis (FEA). The Angle α was altered by modifying condylar position and the inclination of mandibular plane (MP) and occlusal plane (OP) based on the FEM to analyze condylar stress loading under different working conditions. There were differences in anatomical parameters among the different groups, with the smaller Angle α in the osseous destruction group. Angle α was negatively correlated with the inclination of MP and OP. The FEA illustrated condylar stress loading changed after modifying the Angle α by both two modalities. After modifying condylar position, the stress increased with the proximal movement of the condyle toward the OP. After changing the inclination of MP and OP, the stress increased with increasing inclinations. Changes in CHO correlate with condylar osseous condition, and distal movement of the condyle to the OP and reduction of MP and OP inclination may reduce TMJ stress overload. In clinical practice, it is advisable to assess patients for sufficient CHO ratio, as insufficiency in CHO may elevate the risk of TMJ stress overload. The CHO ratio could be modulated by changing the inclination of the OP.

## Introduction

The temporomandibular joint (TMJ) serves as the cornerstone for mandibular movement and adapts to biomechanical stresses to a certain extent^1,2^. Continuous and excessive stress acting on the joint beyond its adaptive capacity may induce destruction, dysfunction, and alterations to its mechanical properties, resulting in temporomandibular disorder (TMD)^3^.

Previous studies have revealed the influence of craniofacial morphology on TMJ stresses, revealing that different vertical growth patterns may affect the load distribution in the joint, and steeper mandibular plane angles and shorter mandibular ramus length may result in higher joint compressive stresses^4–7^. Mandible position abnormalities, such as clockwise rotation of the mandible and retrognathic appearance, correlate with TMJ degenerative disorders^8–10^. A three-dimensional (3D) finite element study found that mandibular deformity may also induce TMJ stresses by affecting the condylar position within the articular fossa^11^. In addition, investigations into occlusal plane (OP) inclination revealed increased inclination may contribute to TMJ stress overload^12–14^.

**Fig. 1:**
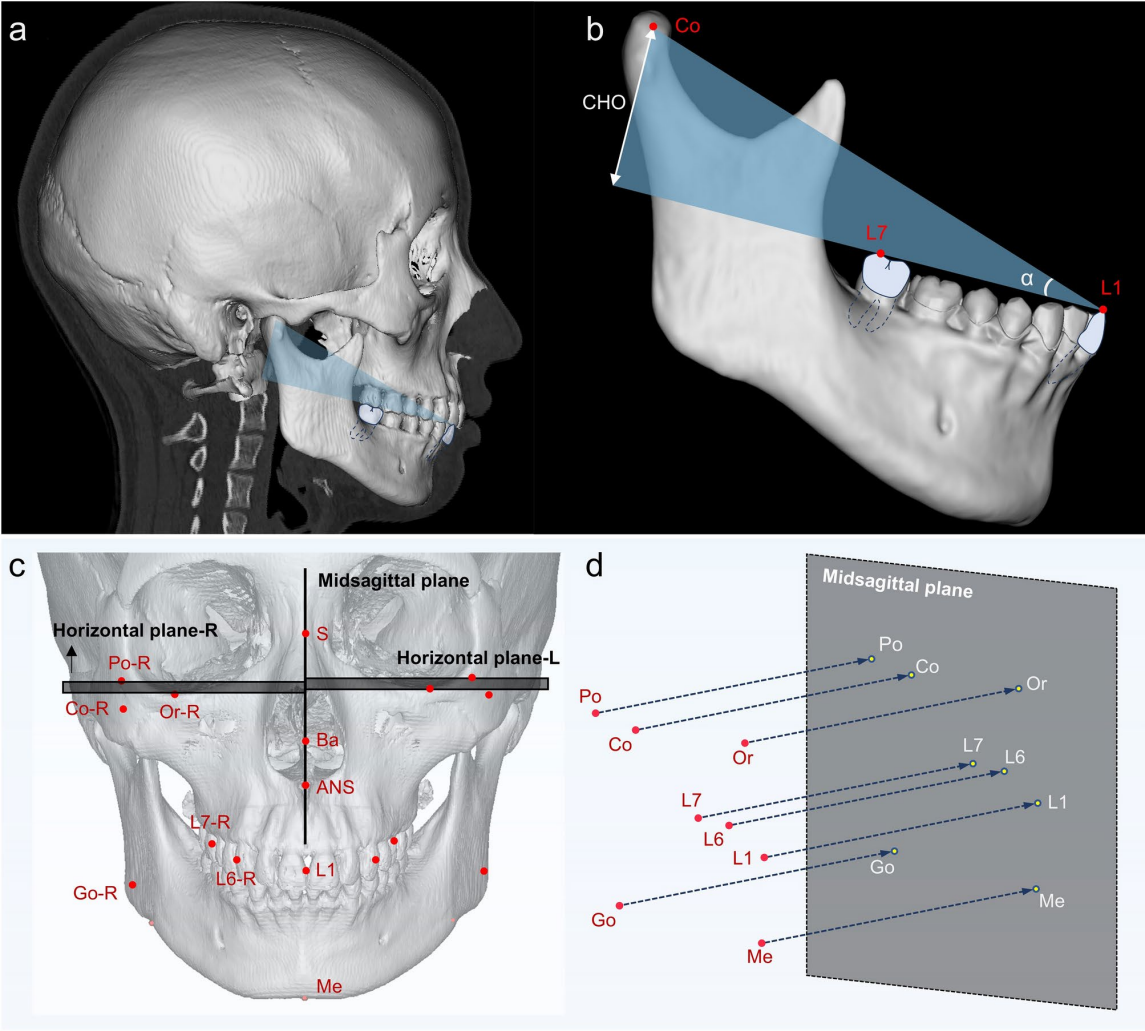
Schematic representation of the three-dimensional (3D) stomatognathic (3DS) model reconstruction and 3D measurements. (**a**) The 3DS model was constructed using the Mimics software. (**b**) CHO represented the vertical height from the condyle to the occlusal plane, and the magnitude of Angle α represents the proportion of CHO. (**c**) Localization of anatomical landmark points on the 3DS model, and create the midsagittal plane and bilateral horizontal planes. (**d**) Anatomical marked points at actual spatial locations (highlighted in red) were projected onto the midsagittal plane as projection points (highlighted in blue).

TMJ stress overload may be associated with condylar bone destruction; however, to the best of our current knowledge, the key factors which regulate this process and the threshold past which condylar bone destruction occurs have yet to be addressed. The DPO (distance to plane of occlusion) value, first proposed by Orthlieb^15^, relates the occlusal plane to the TMJ in an attempt to explain the growth patterns of the jaws and dentition corresponding to different DPO values. Recent evidence indicates that condylar height relative to the occlusal plane (CHO), a variable that corresponds to the DPO value, provides insights into mandibular growth trends and the inclination of the OP^16,17^. A previous 3D theoretical model analysis of this moderating factor demonstrated a trend that the force on the TMJ receptivity decreased progressively with increasing CHO^18^. CHO is jointly influenced by mandibular morphology and occlusal conditions, potentially serving as a pivotal factor contributing to TMJ stress overload (Fig. 1a,b). Modifying mandibular morphology or condylar position to adjust CHO without surgical intervention poses challenges; however, identifying occlusal compensation mechanisms to alter the proportion of CHO for alleviating TMJ stress overload holds promise. However, comprehensive exploration of condylar osseous condition, TMJ loading, and their links with varying proportions of CHO is lacking. Understanding the implications may be useful for risk assessment of TMJ overload in dental practice and also for modulating the TMJ stress environment to reduce the risk of structural damage to the TMJ.

To clarify the impact of CHO and gain deeper insights into the mechanism by which CHO modulates TMJ loading, the present study evaluated the correlation between CHO, related jaw anatomical parameters and condylar osseous condition, as measured by locating anatomical landmarks in 3D space using computed tomography (CT). In addition, a 3D stomatognathic (3DS) model of one patient was constructed based on the 3D finite element method, and the proportions of CHO of the 3DS model were altered from different directions to analyze the changes in TMJ stress overload induced by doing so.

## Methods

### Selection of participants

All data collection and procedures for this study were approved by the ethics committee of the West China Hospital of Stomatology Institutional Review Board (approval number: WCHSIRB-D-2022–364). The study followed the recommendations of the Declaration of Helsinki. All participants provided informed consent to participate in the study and for the publication of their anonymized case details and images.

The anonymous CT data (120 kV, 300 mA, 1 mm slice thickness, and 0.49 mm^3^ voxel size) were acquired from patients who underwent TMJ assessment in the Department of TMJs between October 2018 and August 2022.

The inclusion criteria of participants were: (1) patients diagnosed with TMD according to the Diagnostic Criteria for Temporomandibular Disorders (DC/TMD)^19,20^; (2) patients with permanent dentition; (3) patients aged 18 years and above and under 40 years; (4) patients with established first permanent molar occlusion. The exclusion criteria were: (1) patients with a history of orthodontic treatment, plastic or craniofacial surgery; (2) patients with a history of severe dental or periodontal tissue disease, loss or restorative treatment of multiple teeth (≥ 3); (3) patients with a history of cleft lip and palate, or other systemic diseases affecting craniofacial growth and development; (4) patients with incomplete lateral cephalograms or CT images.

### 3D data measurements

#### Localization of the anatomical landmarks

The CT image data in DICOM format was processed using Mimics Research software (Version 20.0; Materialize, Leuven, Belgium), and a 3D stomatognathic model was reconstructed for each patient (Fig. 1a). The midsagittal plane was constructed using three cranial anatomical points: the Sella (S) point, the Basion (Ba) point, and the Anterior nasal spine (ANS) point^21^; and the horizontal plane, defined for the left and right sides, was the plane perpendicular to the midsagittal plane and passing through the Orbitale (Or) point and Porion (Po) point (Fig. 1c). Anatomical landmark points were defined as in Table 1 and located using the multiple planar reformat tool. The coordinates (x, y, z) of each point were exported for subsequent calculation. The x-direction was defined as parallel to the midsagittal plane and the horizontal plane on each side, with the positive value of the x-coordinate toward the front. The y-direction was defined as perpendicular to the midsagittal plane, with the positive value of the y-coordinate toward the right. The z-direction was defined as parallel to the midsagittal plane and perpendicular to the horizontal plane on each side, with the positive value of the z-coordinate upward.

**Table 1:** Anatomical landmarks and parameters applied in three-dimensional measurement.

Abbreviation	Definition
Landmarks	
Skeletal landmarks	
S	Sella, the center point of sella turcica
Ba	Basion, the middle point of the anterior margin of the foramen magnum
ANS	Anterior nasal spine, tip of anterior nasal spine
Po	Porion, the uppermost point of the external auditory canal
Or	Orbitale, the lowest point of the inferior orbital margin
Me	Menton, the lowest point of the chin
Co	Condylion, the center point of the fitting of the condylar head
ACo	Anterior Co, the most anterior point of the condylar head
PCo	Posterior Co, the most posterior point of the condylar head
SCo	Superior Co, the most superior point of the condylar head
Go	Gonion, the most prominent point of the mandibular angle
Dental landmarks	
L1	The mandibular central incisor edge
L6	The mandibular first molar mesial buccal cusp.
L7/L8	The mandibular last molar with occlusal contact (second or third molar) distal buccal cusp.
Parameters	
CHO	The vertical distance from the Co point to occlusal plane (OP). The OP was drawn perpendicular to the midsagittal plane and passed through L7/L8 and L1 points
L (length)	The horizontal distance from the Co point to OP
Anterior L ratio (ALR)	With L7/L8 points as the boundary, L is divided into the anterior and the posterior. The ratio of the anterior half to the full L
Angle α	Angle between the Co—L1 plane and occlusal plane. The Co—L1 plane was drawn perpendicular to the midsagittal plane and passed through Co and L1 points
FH-MP	Angle between the Frankfort horizontal (FH) plane and mandibular plane (MP). The FH plane was drawn perpendicular to the midsagittal plane and passed through Or and Po points, and the MP plane was drawn perpendicular to the midsagittal plane and passed through Me and Go points
FH-OP	Angle between the FH plane and OP plane
FH-MOP	Angle between the FH plane and molar OP (MOP) plane. The MOP plane was drawn perpendicular to the midsagittal plane and passed through L6 and L7/L8 points
AJS	The anterior temporomandibular joint (TMJ) space, measured on two-dimensional (2D) tomographic images in the sagittal and horizontal planes, was the shortest distance from the ACo point to the articular fossa
PJS	The posterior TMJ space, measured on 2D tomographic images in the sagittal and horizontal planes, was the shortest distance from the PCo point to the articular fossa
SJS	The superior TMJ space, measured on 2D tomographic images in the sagittal and horizontal planes, was the shortest distance from the SCo point to the articular fossa

#### Measurement of the anatomical parameters

Ten anatomical parameters were selected, including the CHO, L, ALR, Angle α, FH-MP angle, FH-OP angle, FH-MOP angle, AJS, PJS, and SJS, and the ultimate angle was measured by projecting onto the midsagittal plane. The abbreviations and definitions of the above parameters are given in Table 1, and the projection methods and measurements are shown schematically in Fig. 1d. To exclude the effect of absolute mandibular size, a relative index—Angle α—was constructed to represent the change in the relative proportion of CHO.

**Fig. 2:**
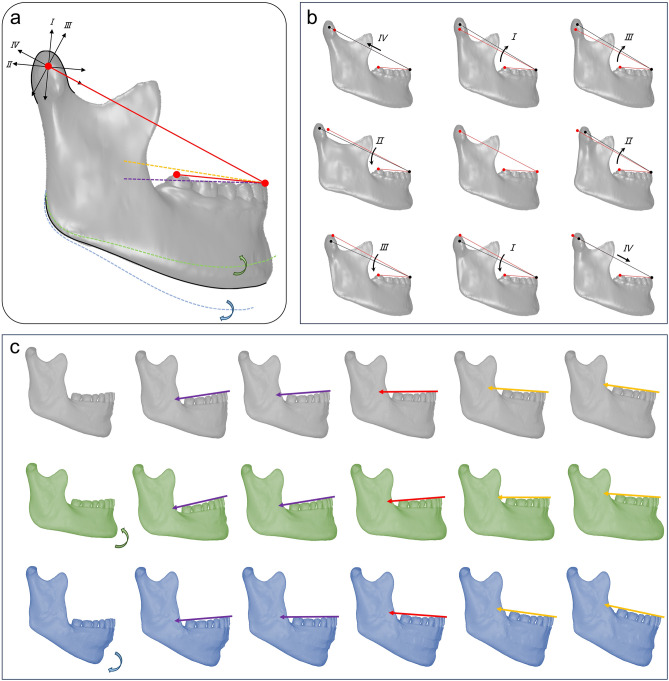
Modifications of Angle α for the three-dimensional stomatognathic (3DS) finite element model. (**a**) Schematic of modifications of the condylar position, the FH-MP angle, and the FH-OP angle. Simulations of modifying the condylar position from four directions: I, II, III, and IV. Simulations of modifying the FH-MP angle from two directions: clockwise (highlighted in blue) and counterclockwise (highlighted in green) around the mandibular angle as the rotation center. Simulations of modifying the FH-OP angle from two directions: clockwise (highlighted in yellow) and counterclockwise (highlighted in purple) around the mesial point of the mandibular central incisor as the rotation center. (**b**) The mandibular model after modification of the condyle positions. (**c**) The mandibular model after modification of the FH-MP angle and FH-OP angle.

All measurements and calculations were performed separately on the left and right sides, to avoid bias caused by facial asymmetry or deformity. The coordinates (x, y, z) of each marked points were obtained in Mimics software. Vector operations were used to obtain the angular measurement projected into the midsagittal plane by substituting the marked point coordinates into the formula. Let $\overrightarrow{n}$ represent the normal vector of the midsagittal plane, and the two vectors expressed the measured angle (β) be $\overrightarrow{a}$ and $\overrightarrow{b}$, calculated as follows:

1 $$\overrightarrow{n}=\left(\begin{array}{c}{n}_{x}\\ {n}_{y}\\ {n}_{z}\end{array}\right)=\left(\begin{array}{c}\left[\left({n}_{y}^{Ba}-{n}_{y}^{S}\right)\left({n}_{z}^{ANS}-{n}_{z}^{S}\right)-\left({n}_{z}^{Ba}-{n}_{z}^{S}\right)\left({n}_{y}^{ANS}-{n}_{y}^{S}\right)\right]\\ \left[\left({n}_{z}^{Ba}-{n}_{z}^{S}\right)\left({n}_{x}^{ANS}-{n}_{x}^{S}\right)-\left({n}_{x}^{Ba}-{n}_{x}^{S}\right)\left({n}_{z}^{ANS}-{n}_{z}^{S}\right)\right]\\ \left[\left({n}_{x}^{Ba}-{n}_{x}^{S}\right)\left({n}_{y}^{ANS}-{n}_{y}^{S}\right)-\left({n}_{y}^{Ba}-{n}_{y}^{S}\right)\left({n}_{x}^{ANS}-{n}_{x}^{S}\right)\right]\end{array}\right)$$

2 $$\overrightarrow{a}=\left({a}_{x}, {a}_{y}, {a}_{z}\right)$$

3 $$\overrightarrow{b}=\left({b}_{x}, {b}_{y}, {b}_{z}\right)$$

4 $$\upbeta =\text{arccos }\left|\frac{\left[\overrightarrow{a}-\left(\frac{\overrightarrow{a}\cdot \overrightarrow{n}}{\left|\overrightarrow{n}\right|}\right)\frac{\overrightarrow{n}}{\left|\overrightarrow{n}\right|}\right]\cdot \left[\overrightarrow{b}-\left(\frac{\overrightarrow{b}\cdot \overrightarrow{n}}{\left|\overrightarrow{n}\right|}\right)\frac{\overrightarrow{n}}{\left|\overrightarrow{n}\right|}\right]}{\left|\overrightarrow{a}-\left(\frac{\overrightarrow{a}\cdot \overrightarrow{n}}{\left|\overrightarrow{n}\right|}\right)\frac{\overrightarrow{n}}{\left|\overrightarrow{n}\right|}\right|\left|\overrightarrow{b}-\left(\frac{\overrightarrow{b}\cdot \overrightarrow{n}}{\left|\overrightarrow{n}\right|}\right)\frac{\overrightarrow{n}}{\left|\overrightarrow{n}\right|}\right|}\right|$$

where ${n}_{x}$, ${n}_{y}$, and ${n}_{z}$ are the x, y, and z components of $\overrightarrow{n}$ respectively; and ${n}^{S}$, ${n}^{Ba}$, and ${n}^{ANS}$ are the coordinates of S, Ba, and ANS point composing the midsagittal plane. As shown in Fig. 2h–k, the angular measurements were visualized.

### Imaging evaluation and grouping

#### Evaluation of condylar osseous condition

The osseous changes of the condyle were assessed and scored in terms of bone surface flattening, bone erosion, osteophytes, bone sclerosis, and bone cysts according to the CT images^22^. The first three items were scored on a scale of 0–3 and the last two items were scored on a scale 0–2^23^. The condylar osseous condition was diagnosed and classified into the following categories^19^:

1. Normal: Normal relative size of the condylar head, and absence of the above-mentioned osseous changes.
2. Indeterminate: Normal relative size of the condylar head, and presence of bone surface flattening and/or bone sclerosis without other observed osseous changes.
3. Osseous destruction (OD): The presence of at least one of bone erosion, osteophytes, and bone cysts with/without other osseous changes were observed.

### 3D Finite element analysis

#### Finite element models

A 3DS finite element model was constructed from the data of a patient with normal condylar osseous condition and morphology. In addition to CT data, the digital dental model and Magnetic Resonance Imaging (MRI) data (3D scanning mode, slice thickness of 1.0 mm, TR: 3200.0, TE: 411.0) were collected for this patient. Initial STL models of the dentition, mandible, maxilla and articular disc were reconstructed using Mimics software according to the patient’s anatomical characteristics (Fig. 1S in the Supplementary Material)^24,25^. All initial models were imported into Geomagic Studio software (Raindrop, North Carolina, America) for smoothing and repair. A circle of periodontal membrane was arranged at the junction of the teeth and jawbone (Fig. 1S in the Supplementary Material), and the thickness of the periodontal membrane was set as 0.2 mm. The right side of mandible and maxilla were excised along the sagittal plane leaving only the left side. Modification of Angle α was simulated in two ways, including changing the condylar position and changing the FH-MP angle and the FH-OP angle (Fig. 2a).

1. The condylar position was moved in the following four sagittal directions by modifying the Angle α. A total of 9 models for computation were obtained (Fig. 2b). Temporal bone and articular disc were moved according to the movement of condyle.
  Setting I Changing the CHO in a direction perpendicular to OP; the Angle α value was increased/decreased by 3°, while the L (length) value remained invariant.
  Setting II Changing the CHO in a direction parallel to OP; the Angle α was decreased/increased by 3°, with the L (length) value increased accordingly by 13.78 mm or decreased by 10.87 mm.
  Setting III Changing the CHO in a direction perpendicular to the connecting line between Co and L1; the Angle α was increased/decreased by 3°, with the L (length) value changed accordingly by 1.87 mm.
  Setting IV Changing the CHO in a direction parallel to the connecting line between Co and L1; the d (diameter) was increased/decreased by 5%, with the L (length) value changed accordingly by 4.12 mm.
2. By modifying the FH-MP of the 3DS model, three basic models were established with the vertical growth patterns of hypodivergent, normodivergent and hyperdivergent facet types^26^. Based on the basic models, the occlusal plane inclination of the models was modified according to the FH-OP of -7–9° (hypodivergent group), -3–13° (normodivergent group), and 1–17° (hyperdivergent group) (Fig. 2c).

Modified models were imported in to 3-Matic Research software (Version 12.0; Materialize, Leuven, Belgium) for dividing the finite element mesh (FEM). The mesh of the entire model was designed as a modified 10-node quadratic tetrahedron element (C3D10M). The mesh size of contact surfaces was refined enough for convergence requirements. The material properties of four components in FEM shown in Table 2^27–29^. In total, the model had 3,896,926 elements and 3,400,348 nodes.

**Table 2:** The material properties of four components in finite element mesh.

	Young’s modulus (MPa)	Poisson’s radio
Periodontal membrane^30^	0.69	0.45
Jawbone^31^	13,700	0.3
Disc^31^	44.1	0.4
Teeth^21^	18,600	0.31

#### Loading and boundary conditions

FEM models were imported into ABAQUS software (2020; Dassault Systèmes Simulia Corp., USA) to complete the load and boundary conditions application and calculation. In order to simulate the actual occlusion, six sets of muscle forces were considered with the following force settings: superficial masseter—47.6 N, deep masseter—20.4 N, anterior temporalis—43.7 N, middle temporalis—39.5 N, posterior temporalis—23.9 N, and medial pterygoid—18.9 N^32,33^. The contact relationships of the articular disc with the condyle and articular fossa, and of the upper teeth with the lower teeth, were set to be hard contact with a friction coefficient of 0.001^34^. Two output variables, contact pressure (CPRESS) and contact force (CFN), were derived by ABAQUS software for characterizing the action force between two surfaces^35^. The six degrees of freedom were fixed on the upper surface of the maxilla and temporal bone. Meanwhile, symmetric boundary conditions were set on the symmetrical faces of the mandible and maxilla.

### Statistical analysis

The above anatomical landmark localization and image assessment were done by at least two experienced specialists. Inter-observer and intra-observer assessments were performed using intraclass correlation coefficient (ICC), and selected measurements were re-estimated at two-week intervals to assess the inter- and intra-examiner reliability of the studied measurements. The ICC values for inter- and re-test reliability were greater than 0.75 for all measures (*P* < 0.05). The measured data was conducted using the mean values.

Data were statistically analyzed using IBM SPSS Statistics (v.22; IBM Corp, Chicago, IL), and *p* < 0.05 was considered statistically significant. Descriptive results of quantitative data were expressed as mean ± standard deviation; normality and chi-square tests were assessed using the Shapiro–Wilk test and Levene test. For indicators of continuous variables with normal distribution, one-way analysis of variance (ANOVA) was applied for multiple group comparisons, and SNK tests were used for further post-hoc testing. For indicators of continuous variables without normal distribution or variance, a Kruskal–Wallis test was used.

## Results

Data gathered from 217 patients was analyzed, including 55 males and 162 females, with an average age of 26.94 ± 6.08 years. Within the total 434 TMJs studied, 80 were devoid of OD, 145 were indeterminate for OD, and 209 were positively identified as having OD.

**Fig. 3:**
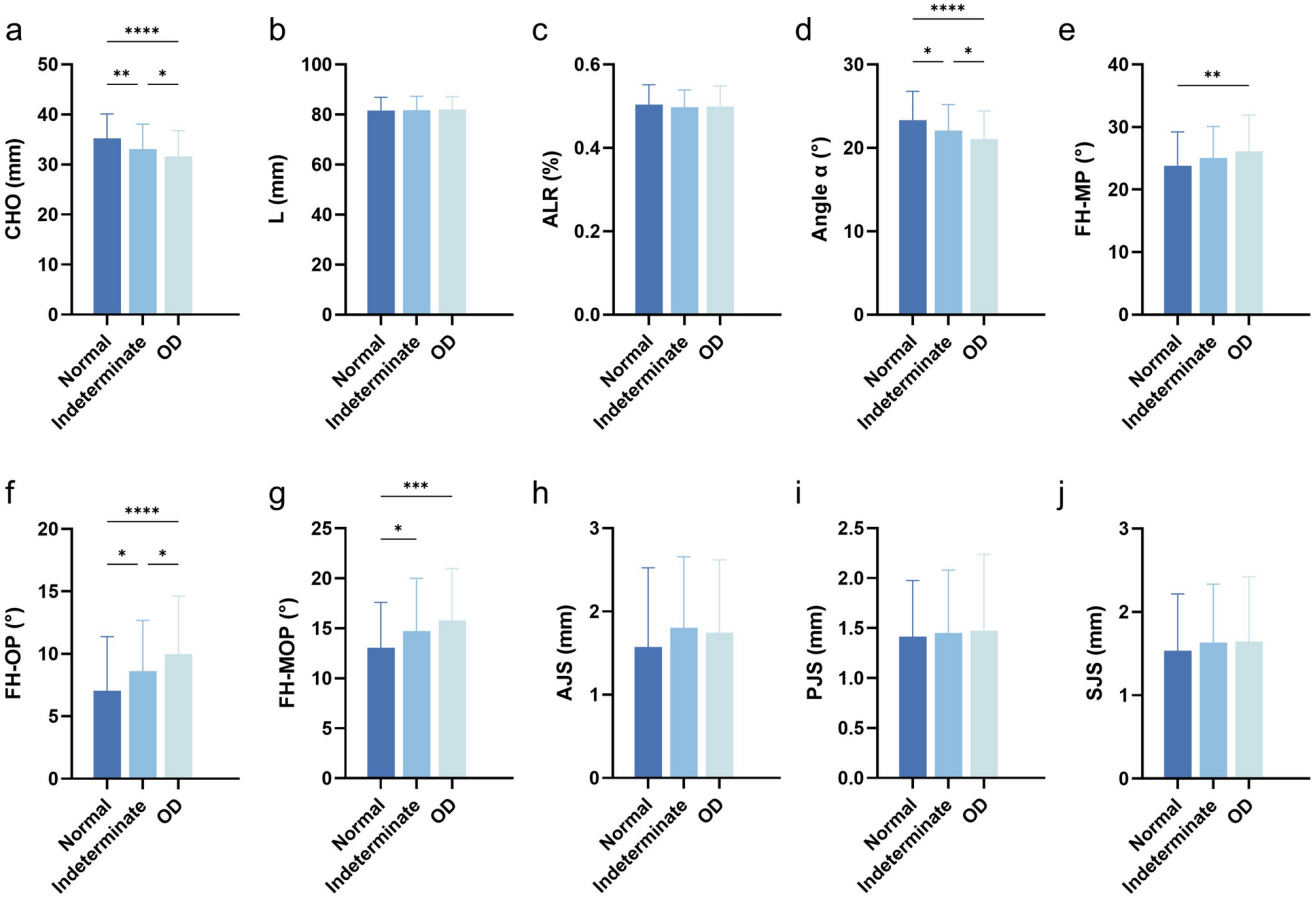
Comparisons of three-dimensional (3D) measurements between groups with different condylar osseous conditions. (**a**) The differences in CHO values among the three groups were significant, with the smallest CHO value in the osseous destruction (OD) group and the largest in the normal group. (**b**, **c**) The differences in L and ALR values among the three groups were not significant. (**d**) The smallest Angle α value was found in the OD group, and the largest in the normal group. (**e**) There was a difference in the FH-MP values between the normal and the OD groups, and the OD group had a larger FH-MP value. (**f**, **g**) The FH-OP values and FH-MOP values in the OD group were significantly greater than those in the other two groups. (**h**, **j**) There were minor differences between the values of the temporomandibular joint space indicators among the three groups. *OD* osseous destruction. *Statistically significant difference at *P* value < 0.05; **Statistically significant difference at *P* value < 0.01; ***Statistically significant difference at *P* value < 0.001; **** Statistically significant difference at *P* value < 0.0001.

The differences in 10 measurement indicators, including CHO, related jaw and teeth anatomical parameters, and TMJ space parameters, were analyzed in different condylar osseous conditions; among them, statistically significant intergroup differences were found in CHO, Angle α, FH-MP, FH-OP, FH-MOP, and AJS (Fig. 3). The values of CHO and Angle α decreased with the increase in the degree of osseous destruction, with the Angle α value was (23.35 ± 3.45)° in the normal group, (22.06 ± 3.13)° in the indeterminate group, and (21.07 ± 3.33)° in the OD group. The normal group had the smallest L value and the largest ALR value, but the differences between the three groups were small and no statistical significances were observed. Among the three groups, the largest values of FH-MP, FH-OP and FH-MOP were in the OD group and the smallest in the normal group, and the distribution of these three variables among the three groups was opposite to the magnitude of the Angle α values. In addition, it was observed that the AJS value in the normal group were significantly smaller than those in the other two groups, and the differences in the PJS and SJS values were not significant.

Table 3 showed the percentage of TMJs with different condylar osseous conditions grouped by Angle α. The number of TMJs with OD were the most in the group with Angle α less than 20°.

**Table 3:** The distribution of temporomandibular joints in different condylar osseous conditions grouped by Angle α.

Condylar osseous condition	N	Angle α (°)				
		< 20	20–22	22–24	24–26	> 26
Normal	80	13	15	24	14	14
Indeterminate	145	33	46	27	24	15
Osseous destruction	209	85	42	42	21	19

**Fig. 4:**
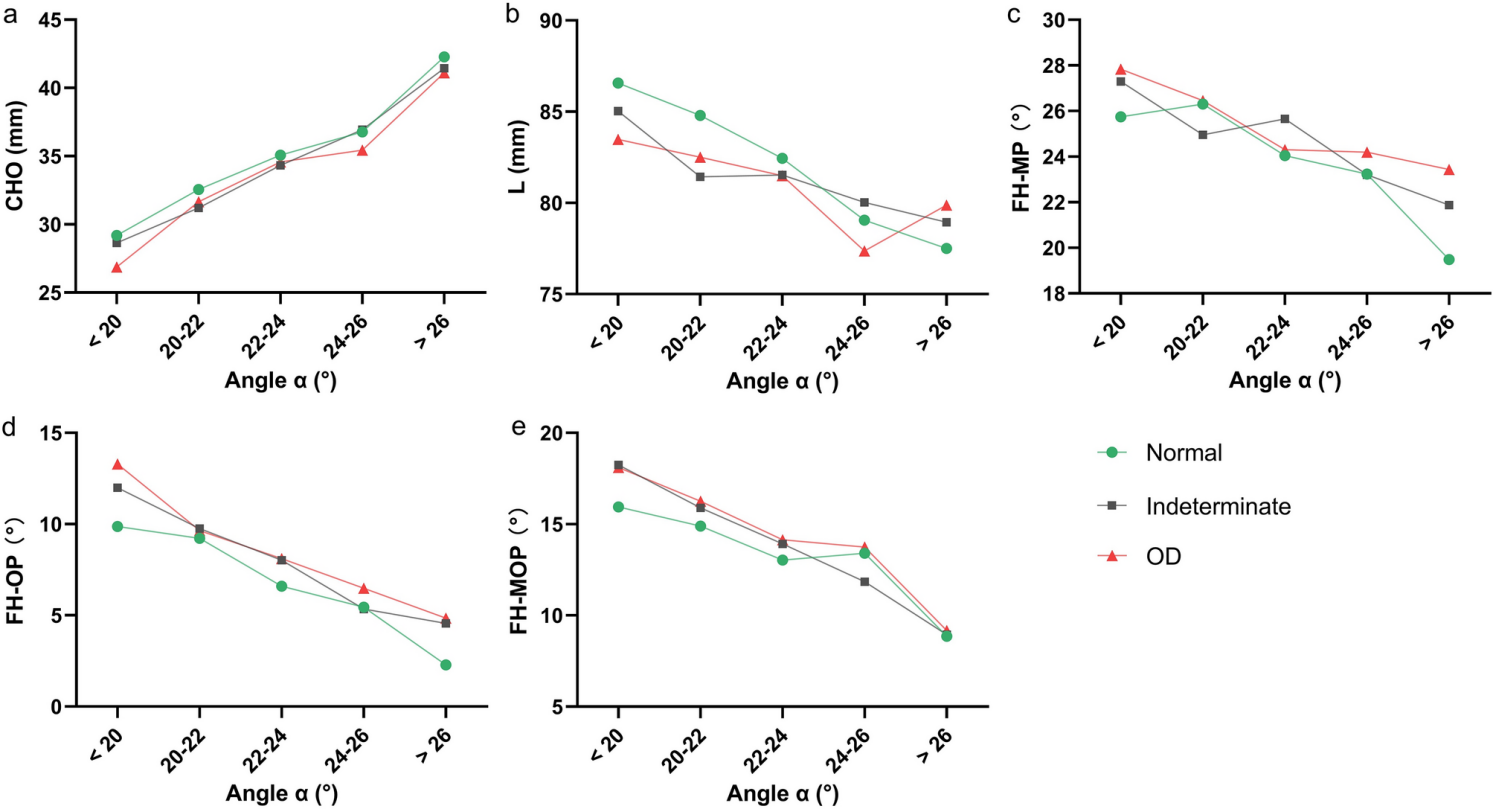
Comparisons of the 3D measured values of anatomical indicators of jaws with different condylar osseous conditions among different Angle α groups. (**a**) The CHO values of all three groups increased with the increase of Angle α. The CHO was small in the OD group and large in the normal group in different Angle α groups. (**b**) The L values of the three groups decreased with the increase of Angle α as a whole, and the L of the OD group was small among the four groups with Angle α of < 26°, while the L of the OD group was the largest among the groups with Angle > 26°. (**c**, **d**) The FH-MP, FH-OP and FH-MOP values of the three groups decreased with the increase of Angle α, and these three values of the OD group were on the large side compared with the other two groups. *OD* osseous destruction.

Further analyses were performed to understand the changes of each measured parameters in different condylar osseous conditions in the different Angle α groups and the changes of jaw and teeth morphology corresponding to different Angle α (Fig. 4). The distribution of variables among subgroups of different condylar osseous conditions showed a consistent pattern as before. Compared with the normal group, the CHO value of the OD group was the smallest, while the FH-MP, FH-OP and FH-MOP values were the largest in different Angle α groups. As the Angle α increased, the CHO values of the three groups increased, while the FH-MP, FH-OP and FH-MOP values decreased.

**Fig. 5:**
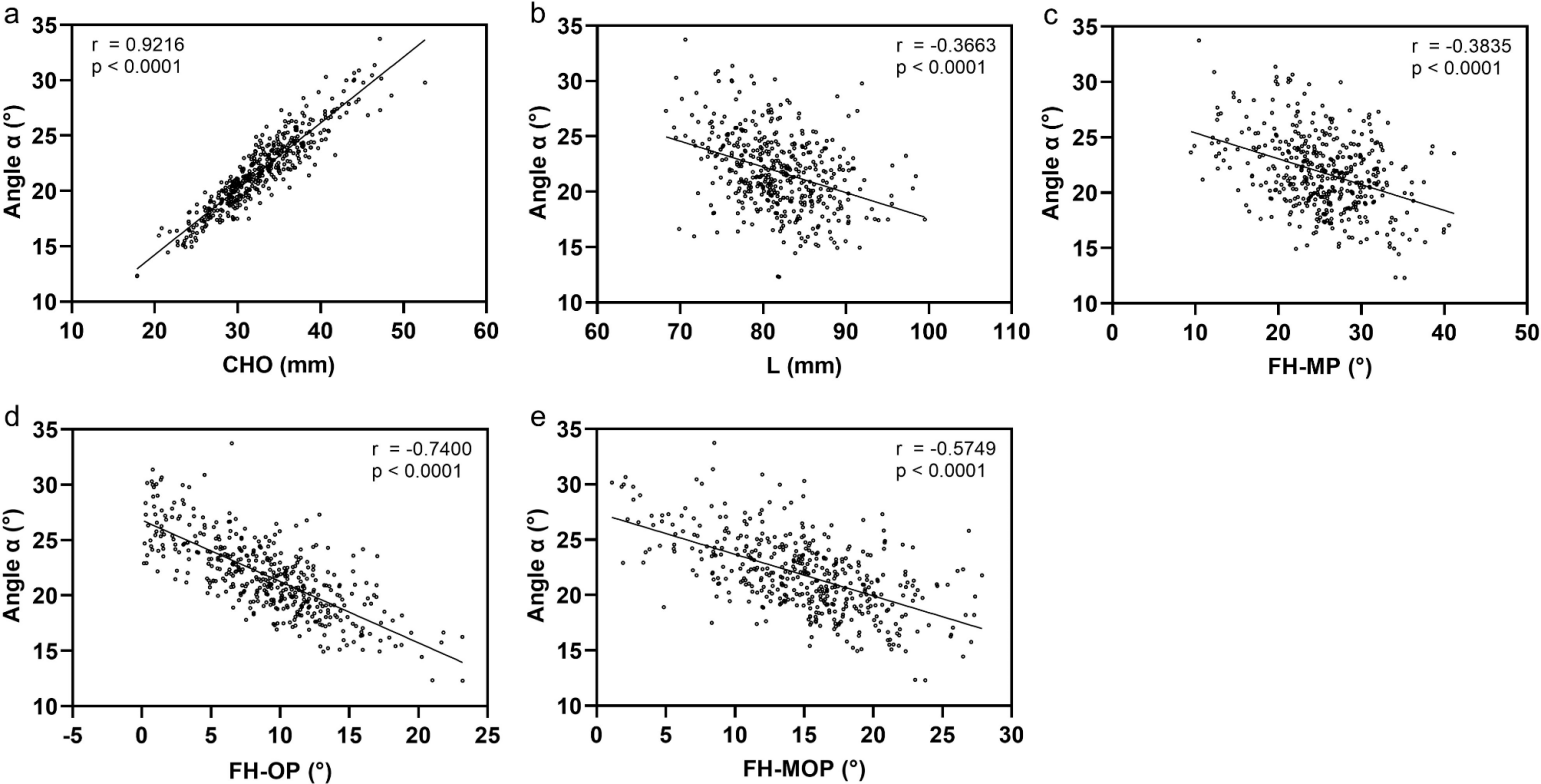
Correlation analysis of Angle α and its associated anatomical indicators of the jaw. (**a**) The CHO value was strongly positively correlated with Angle α, with a class correlation coefficient (r) = 0.9216 (*P* < 0.0001). (**b**) The L value was moderately negatively correlated with Angle α, r = 0.-0.3663 (*P* < 0.0001). (**c**) The FH-MP value was moderately negatively correlated with Angle α, r = 0.-0.3835 (*P* < 0.0001). (**d**) The FH-OP value was strongly negatively correlated with Angle α, r = 0.-0.7400 (*P* < 0.0001). (**e**) The FH-MOP value was moderately negatively correlated with Angle α, r = 0.-0.5749 (*P* < 0.0001).

Analysis of the correlation between Angle α and its related variables showed that the CHO value was strongly positively correlated with Angle α, FH-OP values were strongly negatively correlated with Angle α, while L, FH-MP and FH-MOP values were moderately negatively correlated with Angle α (Fig. 5).

**Fig. 6:**
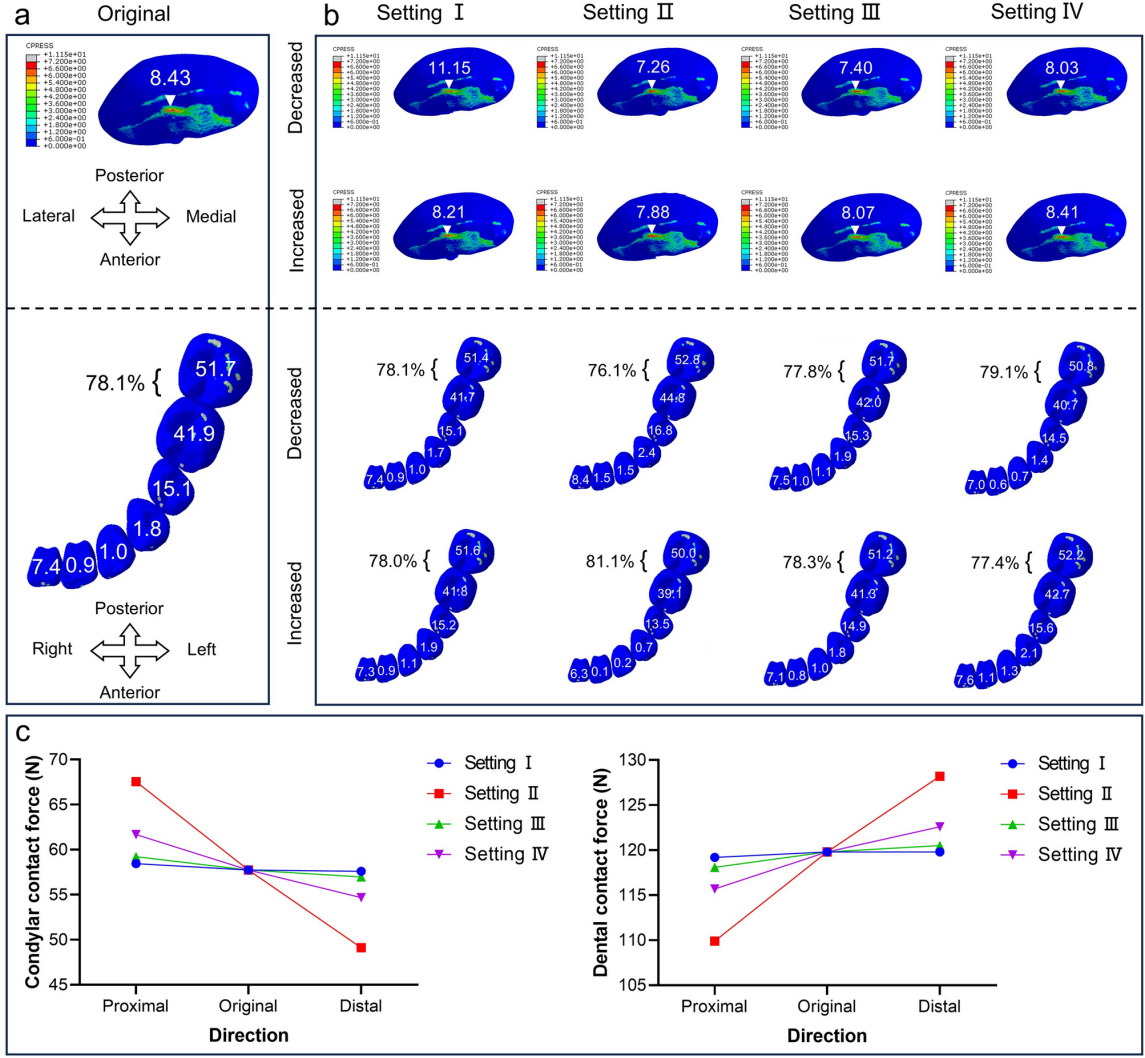
Stress loading results in the condyle and mandibular dentition after moving the condylar position proximal or distal to the occlusal plane of the original model, based on the four settings using the static mechanical analysis methods. (**a**) Stress extremes (in MPa) and stress distribution plots of the condyle before modification, as well as the values (in N) and distribution of CFN on each tooth of the mandibular dentition, and the percentage of CFN in the molar teeth. (**b**) Changes in stress loading of condyle and dentition after modification. (**c**) Trends in the CFN of the condyle and dentition after condylar proximal or distal displacement compared to the original model.

In order to investigate the mechanism of modulating condylar stress loading and mandibular dental forces by changing the condylar position, we modified Angle α in four directions based on 3DS geometrically symmetric model simulations. According to the distribution map of the maximum principal stress of the condyle, the stress distribution regions of the condyle before and after the Angle α changes were mainly concentrated in the anterior oblique plane. Changing Angle α had an effect on the condylar and mandibular teeth stress loading in all four working conditions. Compared with the initial experimental model (Fig. 6a), the stress extremes on the condylar surface decreased accordingly after the condylar positions were moved distally for all the other conditions, except for Setting I, in which the stress extremes on the condylar surface increased after the condyle was moved distally in the direction perpendicular to the plane of the dentition (Fig. 6b). Differences in the distribution of dental forces between the four settings were not significant, with a decrease in the stress distribution on the posterior teeth after the condyle was moved distally in Setting II (Fig. 6b). The trend of contact forces (CFN) on the condylar surface and mandibular dentition for different working conditions is shown in Fig. 6c. As the condyle moved distally to the occlusal plane, the magnitude of Angle α value, and also the proportional composition of CHO/L changed, and the CFN on the condylar surface decreased in all settings, while the CFN on the mandibular dentition increased.

**Fig. 7:**
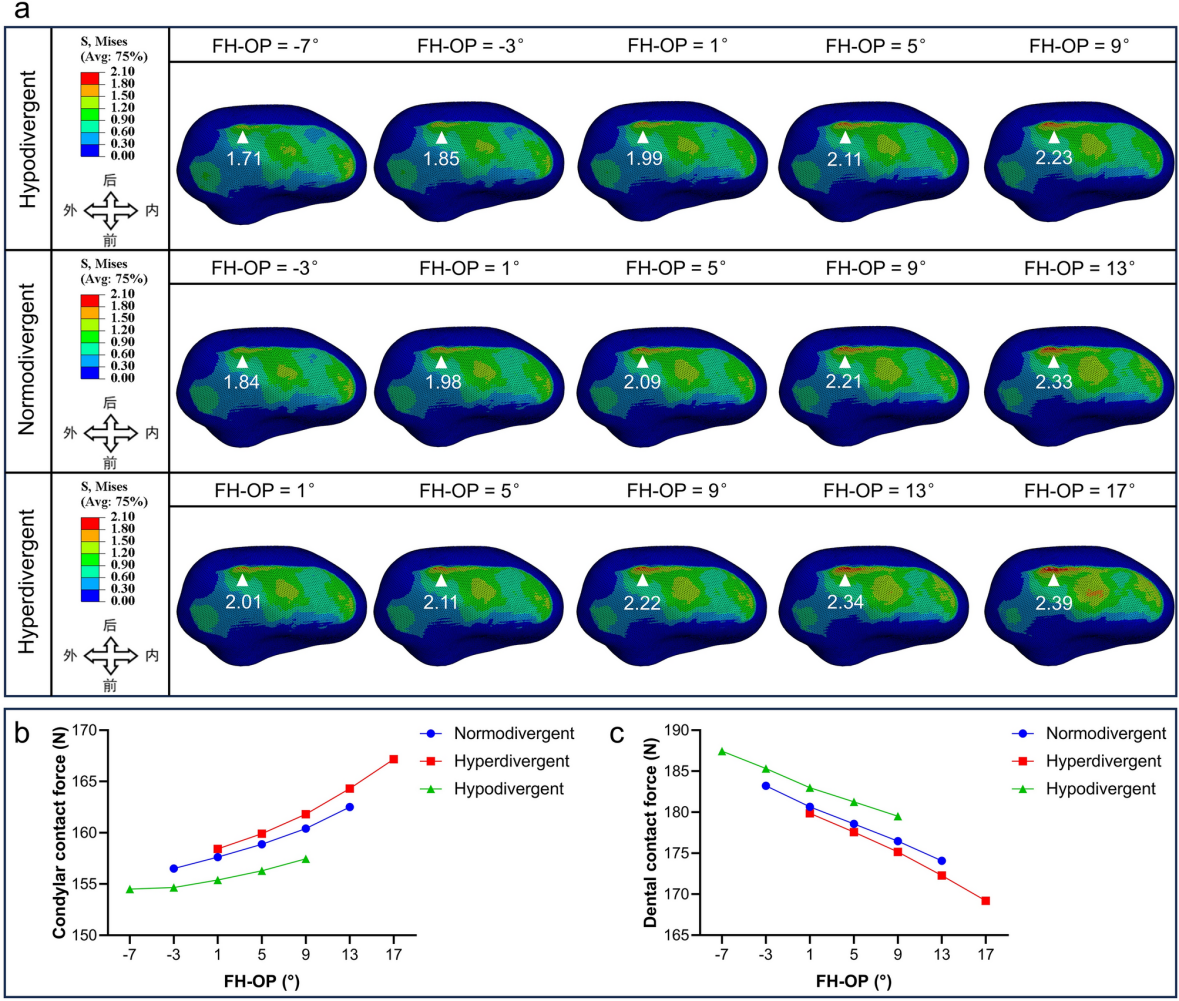
Stress loading results in the condyle and mandibular dentition after changing the FH-MP angle and the FH-OP angle of the original model. (**a**) The von Mises stress nephogram of the condyle after changing the FH-MP angle and FH-OP angle of the original model. The areas of stress distribution in the condyle before and after modification of the model were mainly concentrated in the anterior oblique and parietal surfaces. (**b**, **c**) Trends in the stress CFN of the condyle and dentition. As the FH-OP angle increased, condylar CFN increased and dental CFN decreased in all three groups with different FH-MP. The hyperdivergent group had the largest CFN on the condylar surface and the smallest dental CFN.

Changes in FH-MP or FH-OP can cause corresponding changes in Angle α. In order to observe the effects of changing the above single variables on the condylar stress loading and mandibular dental forces, simulations were performed to modify the FH-OP of experimental models of different vertical growth patterns. Increases in FH-MP and FH-OP values both increased the stress extremes of the condyle, which is consistent with the above measurements of anatomical parameters with different condylar osseous conditions (Fig. 7a). The stress extremes were smallest (1.71 MPa) in the hypodivergent group with FH-OP of − 7° and largest (2.39 MPa) in the hyperdivergent group with FH-OP of 17°. When FH-OP increased by 4°, the condylar extreme stress also increased accordingly. The increase of FH-OP was 0.12 or 0.14 MPa in the hypodivergent group, 0.11–0.14 MPa in the normodivergent group, and 0.05–0.12 MPa in the hyperdivergent group. When the FH-OP angle was constant, the largest stress extremum occurred in the hyperdivergent group. The trend of CFN on the condylar surface was consistent with the change in its stress extremes. With the increase of FH-OP, the condylar CFN value was always the largest in the hyperdivergent group. The change of CFN on the mandibular dentition was opposite to that of CFN on the condylar surface (Fig. 7b,c).

## Discussion

The present study initially investigated the association between CHO ratio and condylar osseous condition and condylar stress loading. The results of the study showed that the CHO ratio can be used as an indicator for assessing the risk of condylar osseous destruction, and the Angle α value provided a reference range for this assessment. Previous studies have demonstrated a consistent trend, namely that mechanical stress on the TMJ varies with individual craniofacial growth patterns as well as with TMJ morphology^36^. Stress overload of the TMJ may cause localized OD or degenerative changes in the condyle^37^. To further validate this relationship, we constructed a 3DS finite element model and performed simulation calculations, which showed that the change of Angle α value would cause the change of condylar stress loading. Previous 3D theoretical model calculations through static equilibrium equations were also consistent with this result^18^.

The traditional method of assessing craniofacial structures and morphology uses lateral cephalograms to measure anatomical indicators in the 2D sagittal plane. However, the overlapping, blurring, and distortion inherent to this imaging modality compromises the validity of analyses based on such measures^38,39^. Dentofacial morphology and characteristics influence the stress distribution of the TMJs bilaterally. Therefore, this study chose to locate the anatomical landmarks on 3D images to establish a stable individual midsagittal plane and to quantify the measurement angles of the unilateral jaws and establish a link with the osseous condition of the ipsilateral condyle. The results indicated that jaws on the side of condylar osseous destruction tended to have a smaller CHO, larger mandibular plane angle, occlusal plane inclination, and molar occlusal plane inclination, which is consistent with the findings obtained in previous studies^9,40^. Finite element analysis provides a preliminary explanation for this phenomenon. The jaw bone with a hyperdivergent growth pattern that has a larger FH-MP angle experienced less stress distributed on the mandibular dentition, but more stress concentrated on the condyle, which may result in osseous destruction of the condyles. The deficiency of CHO and the increase of FH-OP Angle would aggravate this trend of uneven stress distribution^4,14^. These findings are consistent with the results of a previous study on the comparison of bilateral CHO symmetry, where the condyle on the side with a small CHO had a larger CFN^5^. Therefore, relieving condylar stress overload can be considered by increasing the proportion of CHO or decreasing the angle of FH-OP.

**Fig. 8:**
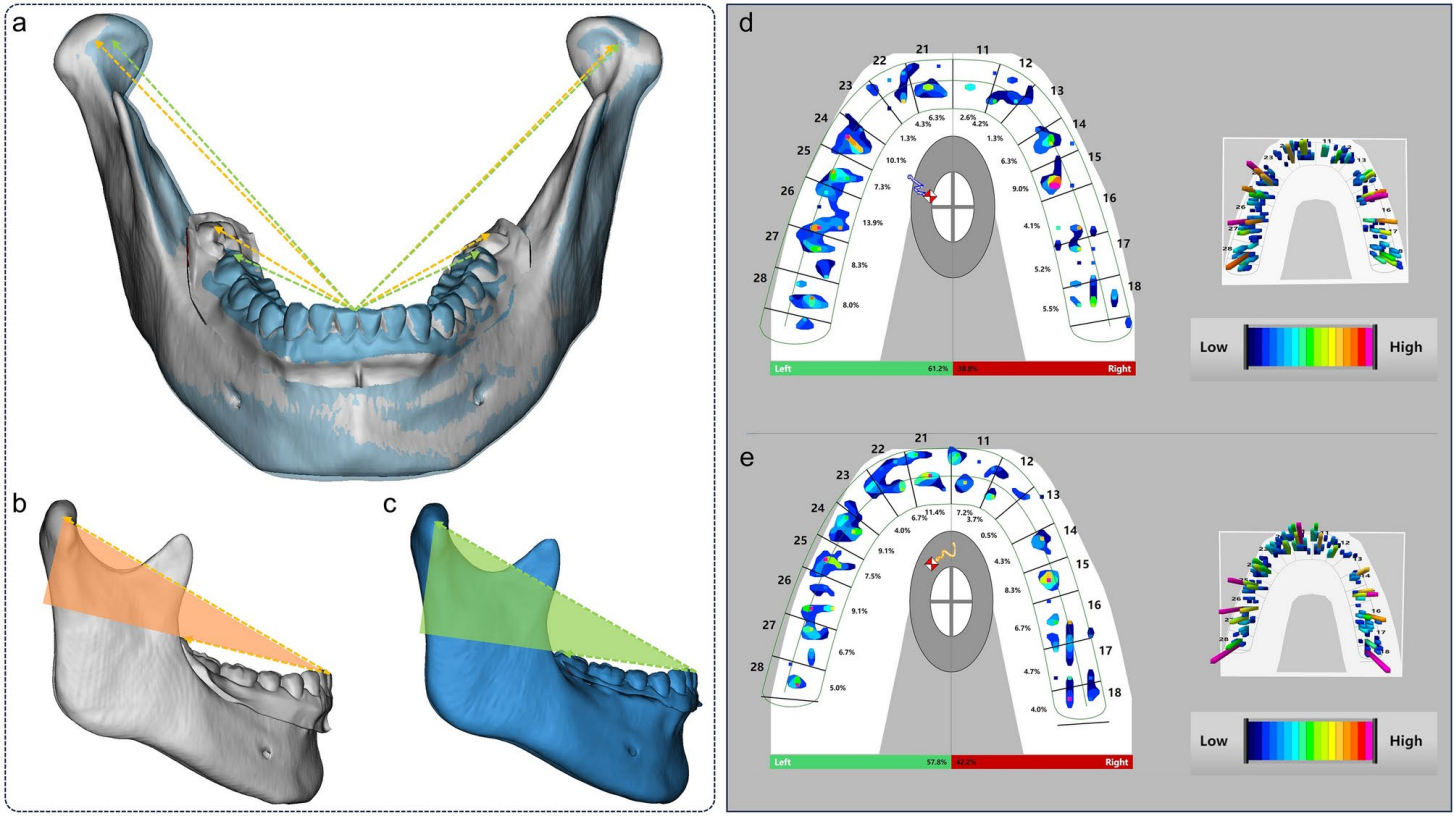
Changes in jaw position and in occlusal forces in patients with third molar extracted before and after extraction. (**a**) Three-dimensional stomatognathic (3DS) models of one patient before and after tooth extraction were reconstructed and registered using the maxillary dentition and intercuspal position (ICP) occlusion to determine the actual changes in jaw position. The white model represents the pre-extraction state, while the blue model represents the post-extraction state. The orange arrow indicates the Angle α before extraction, and the green arrow indicates the Angle α after extraction. (**b**, **c**) Measurements revealed that the Angle α was 17.14° before tooth extraction (highlighted in orange) and increased to 22.63° after extraction (highlighted in green). Following extraction, the mandible rotated counterclockwise, resulting in a decrease in FH-OP and FH-MOP angles and an increase in CHO value. (**d**, **e**) Occlusal force measurements indicated that the occlusal force center shifted anteriorly after tooth extraction, with a more even distribution of bilateral occlusal forces.

The Angle α defined in this study can reflect both the proportion of CHO as well as the change of FH-OP angle and the growth trend of the mandible^16^. Our results also confirmed the correlation between the above relevant anatomical parameters and Angle α indicator. We observed a significant increase in the risk of condylar osseous destruction when the angle α measurement was at or below 21.07°. By adjusting the angle of the FH-OP, intervention in Angle α can be achieved to a certain extent, thus providing an occlusal compensation pathway to reduce the stress loads applied to the condyle and reduce the risk of osseous destruction (Fig. 8a).

Based on the above research findings, we preliminarily observed the clinical symptoms and occlusal force changes in TMD patients who had altered their Angle α clinically by extracting their third molar. The results showed that most patients had improved symptoms of TMJ clicking or pain. Extraction of the third molars with occlusal contact can effectively reduce the value of Angle α, accompanied by alterations in the TMD patient’s jaw position (Fig. 8b,c). Following the extraction, the distribution of occlusal forces became more uniform, and the occlusal center of gravity shifted forward in most of the patients^41^ (Fig. 8d,e). According to a recent study on the occlusal contact and TMJ loading, the forward shift of the occlusal center of gravity was likely to trigger a negative feedback mechanism in the periodontal ligament which in turn reduced the muscle force and hence the TMJ loading^42^. These findings further support the results of this study. Reducing the Angle α value by shortening the dental arch through third molars extraction may achieve adjustment of the stress distribution between the mandibular dentition and condyle.

In addition, the results drawn in the study regarding CHO and Angle α pertain specifically to natural dentitions and have yet to be validated in the context of full-denture prosthodontics. From the perspective of occlusal balance, when providing occlusion to a full denture in the edentulous jaw, reducing the inclination of the occlusal plane is thought to result in occlusal balance and a good prognosis. On the other hand, in the occlusal contacts of the dentate jaw, if appropriate anterior guidance is not provided, there is a possibility that occlusal interference in the molar region will increase.

In this study, the measurements of jaw morphology and finite element simulations were primarily focused on the sagittal plane. Moreover, the simulations of varying jaw morphologies relied on idealized numerical alterations, whereas real cases may not exhibit such extensive numerical fluctuations. Future research could establish asymmetric models and conduct detailed analyses of authentic cases in detail to better explore the connections.

In conclusion, the present study investigated the relationship between CHO and condylar osseous condition and stress loading, and provided Angle α as a reference indicator for risk assessment of TMJ. An excessive proximity of the condyle to the occlusal plane may lead to stress overload and subsequent structural damage of the condyle. A reduction in the inclination of the occlusal plane could be considered to ensure an adequate angle α, thereby balancing the stress distribution between the condyle and mandibular dentition.

## Supplementary Information

Supplementary Figure S1.

## Data Availability

The data underlying this study will be available upon reasonable request to the corresponding author.
