# Supplementary material for: Associations between condylar height relative to occlusal plane and condylar osseous condition and TMJ loading based on 3D measurements and finite element analysis
Source: Sci Rep. 2024 Nov 22;14:28919. doi: 10.1038/s41598-024-80442-x (PMC11582652; doi:10.1038/s41598-024-80442-x)
Supplement: Supplementary file 1 — Supplementary Figure S1. [file 41598_2024_80442_MOESM1_ESM.docx]

**Supplementary Material**


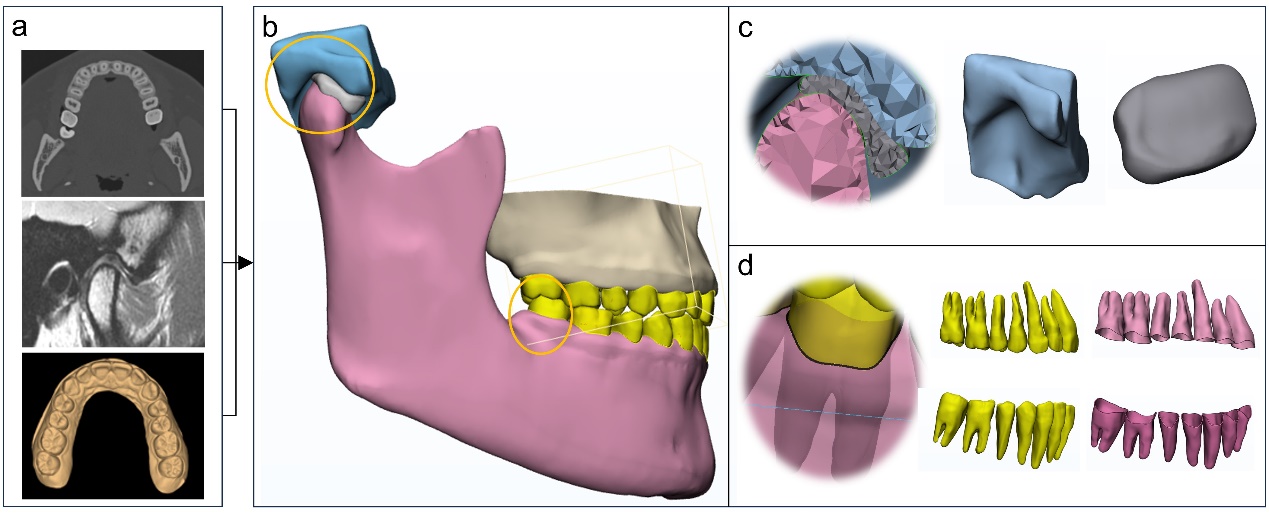


**Figure 1S.** Schematic diagram of the initial three-dimensional stomatognathic finite element model (FEM) construction. **a)** Collection of computed tomography (CT), magnetic resonance imaging (MRI), and digital dentition model data (in order from top to bottom). **b)** Right lateral view of the initial FEM. **c)** Local zoomed-in view of the temporomandibular joint (TMJ) region, including the temporomandibular fossa (blue area) and the articular disc (gray area). **d)** Localized enlarged view of the dentition, including the teeth (yellow area) and periodontal membrane (pink area).
